# Perspective: rheostasis revisited—hibernation and tanycytes

**DOI:** 10.1007/s00360-025-01636-x

**Published:** 2025-11-21

**Authors:** Shona H. Wood

**Affiliations:** Department of Arctic and Marine Biology, Arctic Chronobiology and Physiology Research Group, UiT — The Arctic University of Norway, Tromsø, Norway

**Keywords:** Tanycytes, Rheostasis, Hibernation, Accumulation depletion, Metabolite, Seasonal

## Abstract

Mammalian hibernation is a physiological and behavioural adaptation that permits survival during seasonal periods of energy shortage via a combination of pre-hibernal energy storage and hibernal metabolic depression (torpor). There is both seasonal preparation for the expression of torpor, and the spontaneous termination of hibernation at the end of the season. Small hibernating mammals repeatedly alternate between the torpid state, and the interbout euthermic state over a relatively short timescale (days-weeks) for the entire hibernation season. This is known as torpor arousal cycling (T-A cycling). Hibernation is therefore characterised by extreme shifts in energy homeostasis. Rheostasis is term referring to a change in a regulated homeostatic level or set point. Hibernation can be viewed as rheostasis both over the annual timescale of the seasonal hibernation cycle and over the much shorter T-A cycle. The brain sites through which these homeostatic shifts are controlled have not been identified. A specialised glial cell type lining the 3rd ventricle of the mediobasal hypothalamus (MBH tanycytes), are of particular interest. MBH tanycytes have a privileged anatomical position contacting the periphery and the hypothalamic control centres of the brain. They have documented sensing and signalling function within the hypothalamus, making them a strong candidate cell type for the control of energy homeostasis. Here, I propose that the MBH tanycytes could act as a “rheostat”, shifting their sensitivity to metabolic feedback over the annual timescale and the T-A cycle, and therefore are a promising cell type to investigate in relation to the brain control of hibernation.

## Hibernation – long and short term rheostasis

Homeostatic control in endotherms is a fine balance of maintaining proper physiological function by keeping temperature, pH, nutrient supply and oxygen within set limits. Nicholas Mrosovsky defined the term “rheostasis” (Mrosovsky 1990), to describe examples of physiological phenomenon that behaved as if there was a change in the homeostatic set point. Essentially rheostasis describes a condition in which homeostatic defences are present but there is a change at the level that is defended.

**Fig. 1:**
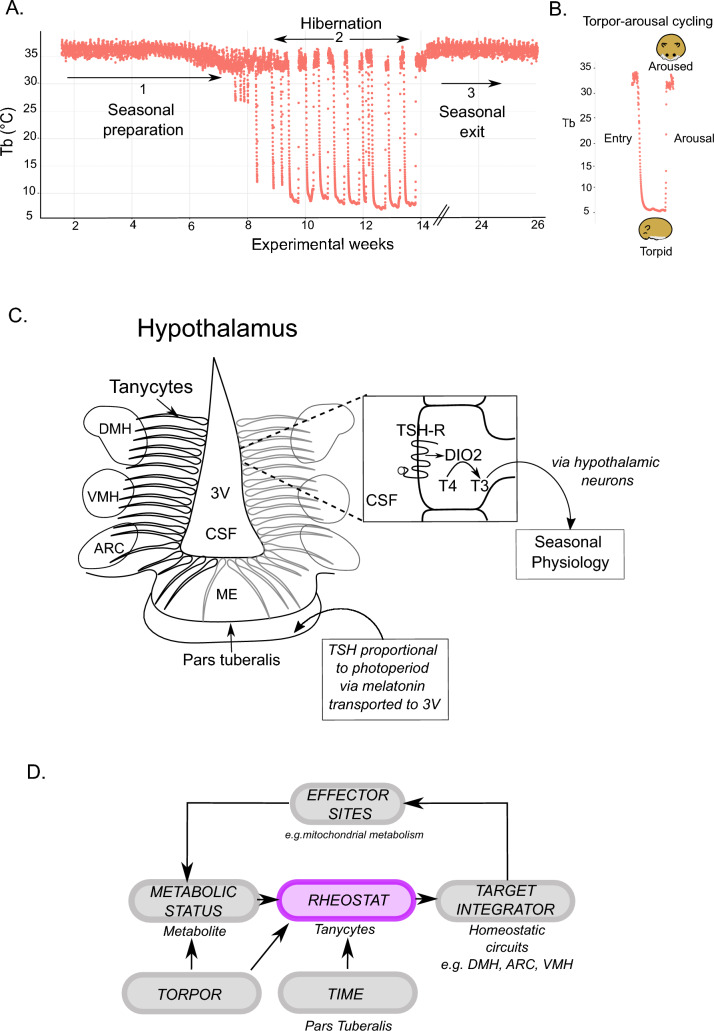
Tanycytes as a rheostat for hibernation. **A** Core body temperature (Tb) of a Golden hamster prior to, during and post hibernation. Animals kept in short photoperiods (8 light: 16 dark) regress their testis and prepare for hibernation (1). Transfer to cold temperatures (7 °C) does not immediately trigger hibernation but does lower Tb from a daily max of approx. 37 °C to 34 °C (1). Test drops in Tb can precede the initiation of hibernation (2), which is characterised by Tb as low as 8 °C during torpor. The seasonal clock leads to the eventual exit from hibernation and reversion to a summer state (3) (Data: Wood et al. unpublished). **B** A zoomed in view of a Torpor – Arousal cycle (T-A cycle) defining the Tb during entry to torpor, and, exit from torpor (arousal) and resumption of (seasonally lowered) euthermy (34 °C). **C**. Privileged anatomical position of tanycytes and the photoneuroendocrine pathway of control of seasonal physiology. *DMH* dorsomedial hypothalamus, *VMH* ventromedial hypothalamus, *ARC* arcuate nucleus, *3 V* 3rd ventricle, *CSF* cerebrospinal fluid, *ME* median eminence, *TSH* thyrotropin, *TSH-R* TSH receptor, *DIO2* deiodinase 2, *T4* thyroxine, *T3* triiodothyronine. **D** Working model for tanycytes as a rheostat receiving metabolic feedback and signalling to the homeostatic circuits which through effector sites alter hibernation physiology, which in turn feedback back on metabolic status. Importantly, “TIME”, received from the *pars tuberalis* is hypothesized to modify the properties of the tanycyte, affecting the interpretation of the metabolic status and altering output signalling, permitting torpor. Torpor itself, likely, alters metabolite status, and subsequently the signalling of tanycytes, resulting in rheostatic function within the T-A cycle

Mrosovsky gave numerous examples of rheostasis including the seasonal body mass cycle in the Svalbard ptarmigan (Mortensen and Blix 1985) and the seasonal regulation of reproduction (Hazlerigg and Simonneaux 2015; Dardente et al. 2019). Here, I focus on hibernation as an example of rheostasis. Hibernation is a seasonally regulated process which results in extreme physiological changes, dramatically reducing metabolic rate to as little as 1% of the active state, lowering core body temperature (T_b_), breathing and heart rate to levels not expected to support life (Lyman et al. 1982; van Breukelen and Martin 2015). These extreme changes in physiological state are even more remarkable because the animal repeatedly cycles between the active (aroused) and hibernating (torpid) states for the entire hibernation season (T-A cycling) (Fig. 1A, B). Throughout hibernation, homeostatic control of body temperature and pCO_2_/pO_2_ is maintained by the brain in both the torpid and aroused states but within widely differing physiological limits (Heller and Hammel 1972; Heller et al. 1974; Lyman et al. 1982).

Deep hibernators, including golden hamsters, ground squirrels and dormice are natural seasonal hibernation models (Lyman et al. 1982; Ruf and Geiser 2015), exhibiting rheostasis on both the short- and long-term basis. First in the seasonal preparative phase for hibernation, with remodelling of the body composition, metabolic rate and T_b_, and in the ultimate spontaneous termination of hibernation in spring. And secondly during T-A cycling, which, depending on the species, sees a change in homeostatic levels over a few days. Therefore, hibernation is an example of rheostasis on a longer-term basis (seasonal preparation and termination of hibernation) and a short-term basis (acute T-A cycle regulation).

In the brain the hypothalamus is likely at the heart of coordinating the physiological adjustments specific to hibernation (Heller and Hammel 1972; Heller et al. 1974). The hypothalamic control of core body temperature, circadian cycle, appetite, body weight, water balance, seasonality, and reproduction are all relevant to the hibernation phenotype. Torpor would challenge many of these regulator systems if the set-points were not altered. For example, a fall in core body temperature from its euthermic temperature set-point (≈37˚C in mammals) is mitigated though the pre-optic area of the hypothalamus, eliciting cutaneous vasoconstriction to limit heat-loss to the environment (Morrison 2016). Torpor, could not occur if this system continued to operate at its euthermic set-point. How set-points are altered, or rheostasis operates, is currently unknown but a mechanism to alter the hypothalamic feedback circuits is of interest. The hypothalamus relies on peripheral feedback via neural responses (example above) but also through hormones/metabolites, these signals must access the brain via fenestrated capillaries or the cerebrospinal fluid (CSF). The seven circumventricular organs (CVO) (Kiecker 2018) are “access” points allowing metabolites/hormones to freely diffuse in and out of the brain. The CSF within the brain ventricles is also of interest in terms of peripheral feedback to the brain. Hibernation involves a suppression of metabolic process throughout the organism, presumably then the regulation of hibernation would consequently require feedback between the brain and periphery. Therefore, tissues or cell types able to sense peripheral feedback and connect to neural circuits of the hypothalamus are of considerable interest in the regulation of energy homeostasis and hibernation.

## Tanycytes – long- and short-term functions

While there are many regions of the brain where neurons and glia contact the CSF or have access to networks of fenestrated capillaries, here I will focus on a specialised glia cell type, tanycytes, which line the 3rd ventricle (3 V) of the mediobasal hypothalamus (MBH). MBH tanycytes have a privileged position, contacting the CSF of the 3 V, and extending processes to a CVO—the median eminence and key hypothalamic sites i.e. dorsomedial hypothalamus (DMH) (Fig. 1C) (Dali et al. 2023). MBH tanycytes are capable of sensing glucose, amino acids, and fatty acids (Frayling et al. 2011; Benford et al. 2017; Lazutkaite et al. 2017; Geller et al. 2019), they also transport leptin and glucose (Balland et al. 2014; Rodríguez-Cortés et al. 2022).

Current theories on the control of hibernation and T-A cycling focus on the accumulation / depletion of specific metabolites/factors driving these cycles (van Breukelen and Martin 2015). MBH tanycytes are therefore an attractive candidate to receive metabolic feedback signals either through the median eminence fenestrated capillaries or the CSF. Metabolic phenotypes are strongly associated to tanycyte function, for example, MBH tanycyte ablation results in increased body fatness (Yoo et al. 2020). Furthermore, alterations in tanycyte vesicle trafficking and tanycyte insulin receptor function results in insulin resistance (Kumar et al. 2021), obesity and glucose intolerance (Duquenne et al. 2021). MBH tanycytes can also signal to hypothalamic neurons (Cortés-Campos et al. 2011; Farkas et al. 2020; Lhomme et al. 2021), and, in one study, optogenetic activation of tanycytes activated feeding responses through signalling via arcuate nucleus neurons (Bolborea et al. 2020). Therefore, MBH tanycyte modulation of metabolic rate and body temperature, via peripheral feedback sensing and hypothalamic neurons, during T-A cycling is hypothetically possible. Recently, we demonstrated that tanycytes express cFOS, a marker of cellular activation, early in arousal from torpor in the golden hamster (Markussen et al. 2024), potentially supporting the idea that tanycytes may receive metabolic feedback from the periphery and signal the arousal process. However, much remains to be done to test this hypothesis, furthermore, the acute sensing role of tanycytes while clearly demonstrated in mice has not been shown in a hibernating model.

On a longer timescale there is strong evidence for tanycyte involvement in the seasonal changes in reproductive status and metabolism which are required for the preparation and termination of hibernation. The MBH tanycytes alter thyroid hormone signalling on a seasonal basis via a well characterised photoneuroendocrine pathway which is synchronised by the seasonally changing photoperiod to entrain a seasonal / circannual (approx. 1 year) clock (Wood and Loudon 2014; Hazlerigg and Simonneaux 2015; Dardente et al. 2019) (Fig. 1C). The changing photoperiod is represented internally by the hormone melatonin which is secreted by the pineal gland during the dark. The melatonin signal is interpreted by the pars tuberalis, a melatonin-receptor (MT1) expressing endocrine tissue sitting between the median eminence of the hypothalamus and the pituitary, relaying seasonal time information to the neighbouring tanycytes via altered production of TSH (Wood and Loudon 2014; Hazlerigg and Simonneaux 2015; Dardente et al. 2019). Tanycytes possess TSH receptors which act to change the amount of Deiodinase enzymes (DIO2 or DIO3) thereby altering active triiodothyronine (T_3_) availability locally within the hypothalamus. The altered thyroid hormone environment results seasonal changes in metabolism and reproduction, likely via RFRP neurons and other hypothalamic neuronal populations (Wood and Loudon 2014; Hazlerigg and Simonneaux 2015; Dardente et al. 2019) (Fig. 1C). Importantly, manipulation of hypothalamic thyroid status blocks or mimics seasonal rheostatic control of physiology, including seasonally prepared daily torpor in Siberian hamsters (Nicholls et al. 1988; Bank et al. 2015, 2017).

Rheostatic function of tanycytes, therefore, could stem from their anatomical position at the nexus point between seasonal time (received as a thyrotropin (TSH) signal from the neighbouring pars tuberalis), metabolic fuel status (received as fuel metabolites via CSF or brain capillaries), and homeostatic control centres in adjacent hypothalamic tissue (received as, yet unknown signals, from the tanycytes themselves). According to this scheme, I hypothesise that the preparation for hibernation involves seasonal TSH-driven changes in MBH tanycyte phenotype, altering the input–output relationship between metabolic fuel signals arriving at MBH tanycytes and tanycyte signals to homeostatic centres of the hypothalamus e.g. DMH, arcuate nucleus, ventromedial hypothalamus (Fig. 1D).

A mechanism to shift the apparent sensitivity of MBH tanycytes (or neurons) to signals is required for this hypothesis. In mice, high fat diet treatments alter the barrier properties of tanycytes (Balland et al. 2014), suggesting that, during torpor, the tanycyte barrier might become “leaky”, altering neuronal sensing of metabolites. However, this is not supported by our preliminary studies (Markussen 2020). Recently, in seasonal maternal photoperiodic paradigm (MPP) in Siberian hamsters, who dramatically alter their body weight and express seasonally prepared daily torpor, we showed that the numbers of sensory cilia on MBH tanycytes alter in response to photoperiod, relating to the overwintering state of the animal (Melum et al. 2024). Siberian hamsters in a MPP also show shifts in the sensitivity to TSH depending on the photoperiod experienced (Saenz de Miera et al. 2017). And, in the hibernation model, the golden hamster, we have observed altered ciliary gene expression in MBH tanycytes relating to seasonal status (Melum et al. 2025). Cilia are scaffolds for cell-surface G-protein coupled receptors (GPCRs) and enhance signalling function (Schou et al. 2015). Furthermore, there are several ciliopathies that are characterised by metabolic phenotypes (Loktev and Jackson 2013), and inhibition of ciliary assembly can reduce neuronal sensitivity to metabolic feedback signals resulting in obesity (Volta and Gerdes 2017). Therefore, it is possible that changes in the number of MBH tanycyte cilia alters sensitivity and feedback to the hypothalamus.

I suggest that a seasonally regulated shift in the sensitivity of MBH tanycytes to metabolic feedback, via altered ciliary signalling, supports the hibernation state. Furthermore, changes in sensitivity may also account for the spontaneous termination of the hibernation season, as ciliary gene expression in MBH tanycytes appears to increase at the termination of the hibernation season (Melum et al. 2025). Therefore, the seasonally modified MBH tanycyte may allow a shift in set point (rheostasis) to metabolic feedback to allow the expression of torpor. This model only explains the seasonal regulation of hibernation as it is not expected that there are alterations to ciliary assembly/numbers within the T-A cycle. However, as a result of the metabolic depression of torpor, body temperature declines, this means that the rate of biological activity of GPCRs and their signalling effects via cilia will decline due to Q10 effects. This decline in biological process rate could be a possible mechanism to shift the apparent signalling set points during the T-A cycle. In line with this, the torpor and interbout bout durations are usually temperature dependent (Ruf and Geiser 2015; Malan et al. 2018). This would fit with an accumulation/depletion model (van Breukelen and Martin 2015) but with the modification that factors accumulating during torpor may alter according to temperature and metabolic rate but that also the sensitivity to these factors could alter depending on the threshold or rate of signalling of MBH tanycytes (Fig. 1D). In this scheme the seasonal regulation of MBH tanycyte sensitivity would be permissive for the entry and exit into the hibernating state and required for the regulation of T-A cycling, while not specifically driving the T-A cycle.

The hypothesis presented here remains to be thoroughly tested. However, the case for focusing on MBH tanycytes is strong; a well characterised role in seasonal physiology, an acute sensing function, and an anatomical position contacting the periphery and the hypothalamic centres. The paradigm of hibernation is an excellent opportunity to probe the function of MBH tanycytes further and potentially reveal the regulatory mechanisms behind this extreme metabolic state.

## Predictions and testability

One assertion of the hypothesis I have put forward is that there is an accumulation or depletion of specific metabolites that are sensed by the tanycytes during T-A cycling. To test this the metabolites in the CSF and the blood plasma must be characterised in an individual animal throughout a T-A cycle to identify candidate metabolites. Then, testing whether these metabolites can be sensed by tanycytes and if they alter the T-A cycle dynamics is needed . This requires careful development of individual animal monitoring, and *in-vivo* micro- and retro- dialysis techniques to sample from the 3rd ventricle. Furthermore, application of slice culture calcium imaging of tanycytes to test responsiveness to candidate metabolites would be informative. Even then, the specific role of MBH tanycytes could be questioned because of the other brain regions and cells contacting the periphery and CSF. To go further, genetic manipulation of deep hibernators is required, fortunately, this is becoming increasingly tractable (Shigeta et al. 2025). Specific, to the presented hypothesis disruptions to MBH tanycyte cilia by targeted deletion of Kif3a or IFT88 should alter seasonal preparation and potentially prevent the permissive state to allow T-A cycling. Manipulation of MBH tanycyte receptors to identified metabolites and demonstrating subsequent effects on T-A cycle dynamics is also an option. As is, optogenetic or chemogenetic activation of tanycytes during torpor to demonstrate a role in the arousal process. Of course, if these studies show no effects on T-A cycling and/or seasonal preparation then the hypothesis presented here can be rejected.
